# Comprehensive integration of mental health screening and triage with mental performance services through a sport science hub model in collegiate sport

**DOI:** 10.1007/s44192-026-00552-y

**Published:** 2026-07-19

**Authors:** Andreas Stamatis, Ernest Rimer, Nathan Wells

**Affiliations:** 1 Department of Health and Sport Sciences, University of Louisville, Louisville, USA; 2 University of Louisville Health, Sports Medicine Institute, Louisville, USA; 3 Athletic Department, University of Louisville, Louisville, USA

**Keywords:** Mental toughness, SMHAT-1, NCAA, Data governance, Collegiate athletes, Implementation science, Integrated care, Organizational design

## Abstract

Collegiate sport environments often bring mental health (MH) and performance (MP) into close interaction; yet, screening, triage, referral, and MP support often remain operationally fragmented. This fragmentation may weaken follow-through after screening, limit coordination across clinical and performance services, and create uncertainty around privacy, scope boundaries, and accountability. In this Perspective, drawing on implementation science, integrated care, and systems-oriented organizational design, we suggest that a promising direction in athlete MH-MP support is not simply expanding screening availability, but building governed infrastructure that better connects identification to action. We propose a sport science hub model for coordinating two linked but distinct lanes: (1) structured MH screening, triage, and clinician-led referral workflows, and (2) MP profiling and skills-based support delivered within non-clinical scope. The model is organized around explicit guardrails, including role separation, privacy-secured and auditable access, non-punitive use protections, and emergency escalation pathways. We discuss the rationale for using structured screening and referral processes while acknowledging the limitations of current screening tools, including false-negative and false-positive concerns, and the importance of interpreting performance disruption in context rather than medicalizing normal variation. We conclude with governance principles and a forward-looking research agenda focused on implementation, trust, acceptability, screening accuracy, and prospective evaluation of integrated MH–MP systems.

## Why mental health–mental performance integration is needed, and what this article contributes

Collegiate athletes operate in environments in which *mental health* (MH) and *mental performance* (MP) are closely intertwined. NCAA Mental Health Best Practices emphasize routine screening, defined referral pathways, qualified provider oversight, and trust protections, including that screening information should not be used to determine playing time, selection, scholarship status, or team standing [1]. These expectations make follow-through as important as identification: screening should connect student-athletes to timely, appropriate, and trusted support.

International sport guidance similarly supports structured recognition, screening, and referral rather than ad hoc responses. The International Olympic Committee developed the Sport Mental Health Assessment Tool 1 (SMHAT-1) and the Sport Mental Health Recognition Tool 1 (SMHRT-1) to support earlier identification, triage, and clinician-led follow-up for athletes who may need care [2]. However, recent analyses have highlighted limitations in screening performance, including false-negative concerns, reinforcing that such tools should be used within a broader governed clinical workflow rather than as stand-alone diagnostic solutions [3, 4]. In collegiate settings, unexplained performance disruption may warrant consideration within MH triage when not better accounted for by injury, illness, training load, or other contextual factors, but should not be treated as evidence of psychopathology in isolation [1, 2].

Despite expanded expectations for athlete MH support, many collegiate systems remain fragmented. Even where “high-performance” structures exist, resource allocation, reporting lines, operational asymmetries, and coach-dependent communication may preserve silos across clinical and performance services, potentially weakening follow-through after screening and limiting coordinated care. This suggests that MH–MP fragmentation is not only an awareness problem, but also an infrastructure and governance challenge [1, 5]. *Comprehensive integration* provides a useful starting point for addressing this fragmentation. Rimer and colleagues describe comprehensive integration as an approach for moving beyond traditional high-performance structures, emphasizing the coordinated use of accessible services and resources to support athlete health and performance [5]. In the MH–MP context, comprehensive integration refers to coordinated infrastructure that connects services while preserving clinical and performance role boundaries, privacy protections, and accountability for referral and follow-up.

This infrastructure and governance argument can be situated within three overlapping literatures: implementation science, integrated care, and systems-oriented organizational design (see Table 1). Implementation frameworks, such as CFIR, RE-AIM, and EPIS, help identify the multilevel determinants of adoption, implementation, evaluation, and sustainment, including athletic department culture, leadership engagement, staffing, technological readiness, policy requirements, privacy constraints, athlete trust, practitioner role clarity, referral workflows, documentation, emergency escalation, and audit procedures [6–9].

**Table 1:** Theoretical lenses supporting the proposed MH–MP coordination framework

Model component	Implementation science	Integrated care	Systems/organizational design
MH screening, triage, and referral	CFIR implementation process; RE-AIM reach and effectiveness	Population-based identification; stepped care	Functional and clinical coordination
Role separation between MH and MP	CFIR individuals and inner setting	Team-based care with defined scope	Professional boundary management
Privacy-secured routing and audit trails	CFIR inner setting; implementation process	Accountable care	Information-system integration
Lane B → Lane A referral pathway	RE-AIM implementation fidelity	Warm handoff; transition management	Coordination across semi-autonomous roles
Emergency escalation and follow-up	EPIS implementation and sustainment	Matched intensity of care	Standardization with local adaptation
Local adaptation across institutions	EPIS exploration, preparation, and sustainment	Context-sensitive care pathways	Complex adaptive systems

This table summarizes how implementation science, integrated care, and systems/organizational design frameworks inform key components of the proposed MH–MP coordination model, including screening and referral, role separation, privacy-secured routing, emergency escalation, and local adaptation across collegiate sport settings. MH = mental health; MP = mental performance; CFIR = Consolidated Framework for Implementation Research; RE-AIM = Reach, Effectiveness, Adoption, Implementation, Maintenance; EPIS = Exploration, Preparation, Implementation, Sustainment

In practical terms, integrated care principles suggest several design requirements for collegiate MH–MP coordination: routine identification, defined referral pathways, role clarity, follow-up monitoring, and accountability for whether concerns move from recognition to action [10]. Stepped-care models similarly support matching the intensity of response to level of need, rather than treating all concerns as requiring the same pathway [11]. Applied to collegiate sport, these principles point to a central design challenge: coordinating non-clinical MP support, clinician-led MH assessment, and emergency escalation without blurring role boundaries or weakening follow-through.

Systems and organizational theory further clarify why MH–MP coordination should be designed as an adaptable workflow rather than a fixed protocol. Athletic departments function as complex adaptive systems in which athletes, coaches, athletic trainers, physicians, licensed MH providers, MP practitioners, sport scientists, administrators, and external providers operate with different incentives, constraints, professional boundaries, and information needs. In this context, integration does not require full structural merger or blended clinical-performance roles. Rather, these perspectives point toward functional coordination through shared workflows, privacy-secured information systems, auditable routing, and clinical coordination through defined referral, handoff, escalation, and follow-up processes [12–14].

Comparable models in healthcare, military, and elite sport reinforce this same design logic. Collaborative care models emphasize screening, care coordination, specialist consultation, measurement-informed follow-up, and stepped referral pathways [10, 15]. Military and embedded behavioral health models similarly link routine screening with care management and referral while highlighting confidentiality and career-impact concerns that parallel athlete fears about privacy, selection, and non-punitive use [16–21]. Sport-specific approaches, including the IOC SMHAT-1/SMHRT-1 framework, USOPC applications of SMHAT-1, and the Canadian Centre for Mental Health and Sport’s collaborative care model, provide further precedents for structured screening, clinician-led follow-up, interdisciplinary collaboration, secure information-sharing, and explicit scope boundaries [2, 3, 22, 23]. Taken together, these examples provide plausible design considerations for collegiate MH–MP coordination: structured identification, governed handoffs, secure information-sharing, clinician-led follow-up, explicit scope boundaries, and ongoing evaluation.

Several organizational arrangements could address MH–MP fragmentation. A separated interprofessional model allows licensed MH providers, MP consultants, athletic trainers, physicians, and sport scientists to retain distinct roles while coordinating through referral pathways and team-based communication. This preserves disciplinary expertise but may reproduce fragmentation if handoffs, documentation, and accountability are weak [23, 24]. A more fully integrated or dual-credentialed model may provide more continuous athlete-facing support, but it depends on the availability of appropriately trained personnel and still requires governance to protect privacy, role clarity, and clinical independence [25–27]. The framework proposed here occupies a “middle ground:” a hub-supported interprofessional coordination model that preserves distinct MH and MP roles while using shared workflow design, referral routing, privacy protections, and accountability mechanisms to connect services across lanes [28, 29].

Building on this middle-ground position, this Perspective proposes a governed MH–MP coordination framework for collegiate sport. The framework links two distinct but connected lanes: (1) MH screening, triage, referral, and clinician-led follow-up, and (2) MP profiling and skills-based support delivered within non-clinical scope boundaries. It is designed to coordinate clinical care and performance support through shared workflow design, explicit governance, role separation, privacy protections, and auditable escalation pathways. Accordingly, this Perspective makes three contributions: it frames scalable MH–MP coordination as, in part, an infrastructure and governance challenge; it proposes an illustrative hub-supported framework that preserves scope boundaries, athlete trust, and clinician-led care; and it outlines a research agenda focused on implementation, acceptability, screening accuracy, workflow performance, and prospective evaluation. Overall, we argue that governed system integration is a promising candidate approach for moving collegiate athlete support beyond fragmented service availability, but one that requires further implementation testing and prospective evaluation.

## The MH–MP comprehensive integration model: sport science as coordinating hub

In this article, sport science is positioned as an integration function that connects workflows across MH and MP. Building on the logic of comprehensive integration, the hub role is to coordinate shared touchpoints, role-specific routing, documentation, and accountability so that screening is more consistently connected to appropriate follow-up rather than remaining a stand-alone administrative exercise [3, 4, 30]. In practice, this can be operationalized through a privacy-secured athlete management platform or comparable workflow system that can support electronic administration of MH and MP measures, automated scoring, role-based routing, and auditable follow-up. This workflow is presented as a proposed coordination framework rather than as a one-site software protocol. The model is organized around the core safeguards described in Sect. 3.1.

Within this model, Lane A is the clinician-led MH lane. Structured screening and triage can be conducted using SMHAT-1-aligned workflows, with the Athlete Psychological Strain Questionnaire (APSQ) serving as the initial triage step and positive findings prompting disorder-specific screening and clinician-led assessment and management as indicated [2]. We use SMHAT-1 as an example of a structured screening and referral pathway because it offers a standardized mechanism for recognition, triage, and follow-up, while acknowledging that screening tools should be embedded within broader clinical workflows rather than treated as stand-alone diagnostic solutions. Recent analyses highlighting false-negative concerns underscore the importance of embedding screening tools within a broader governed clinical workflow, with qualified providers responsible for interpretation, follow-up, risk assessment, and emergency decision-making [1–4].

Lane B is the non-clinical MP lane. It is organized around MP competencies relevant to goal-directed functioning under competitive stress, including attentional control, emotion regulation, coping effectiveness, confidence, concentration, goal setting, self-talk, imagery, and adaptive response to challenge. *Mental toughness* (MT) is used here as an integrative performance construct example within this competency space, not as a synonym for resilience, coping, or self-regulation. Conceptual and empirical work has positioned MT as a psychological resource relevant to sustained goal pursuit and performance under pressure, while also distinguishing it from adjacent constructs through its nomological network and associations with coping, stress appraisal, resilience, performance, goal progress, and thriving under stress [31–34]. Mental toughness training models and mental skills interventions also commonly include processes such as goal setting, imagery, self-talk, emotion regulation, and concentration, aligning MT-informed profiling with applied MP practice rather than clinical assessment [35–37].

The Mental Toughness Index (MTI) was selected for pragmatic, psychometric, and implementation reasons. It is a brief 8-item, accessible measure with evidence for structural validity, internal consistency, and nomological validity across sport and achievement contexts, and its use was supported by existing practitioner expertise within our applied performance environment [34]. Cross-cultural measurement work supports the MTI’s use across multiple athlete samples, including Australian, Chinese, Malaysian, Brazilian, American, and Greek contexts [38–41]. The MTI has also been used in collegiate athlete research examining MT alongside MH-relevant constructs, such as self-compassion, and NCAA MH best-practice implications [42]. In this framework, the MTI is used as an example of a non-diagnostic applied profiling tool to guide skills-based MP support and support-matching within scope. Its use is limited to non-clinical support planning and does not extend to clinical screening, selection criterion, or normative standard of athlete worth.

This use of the MTI separates measurement from cultural enactment. Critiques of MT have noted that sport cultures may define or perform “toughness” through norms that reward distress suppression, harm tolerance, denial of vulnerability, or reduced help-seeking [43–46]. MTI-informed profiling is used only to support non-punitive MP development, contextual discussion, and referral when concerns exceed non-clinical scope.

The connecting function of the sport science hub lies in workflow design, routing, and accountability. The hub coordinates baseline and event-triggered touchpoints, supports routing of results to the appropriate licensed or qualified personnel, and is intended to strengthen documentation, auditability, and closed-loop follow-through. Unexplained performance disruption may sometimes prompt consideration of further screening or referral when not better explained by injury, illness, training load, or other contextual factors, and should be interpreted within a broader clinical and performance context [1, 2].

**Fig. 1:**
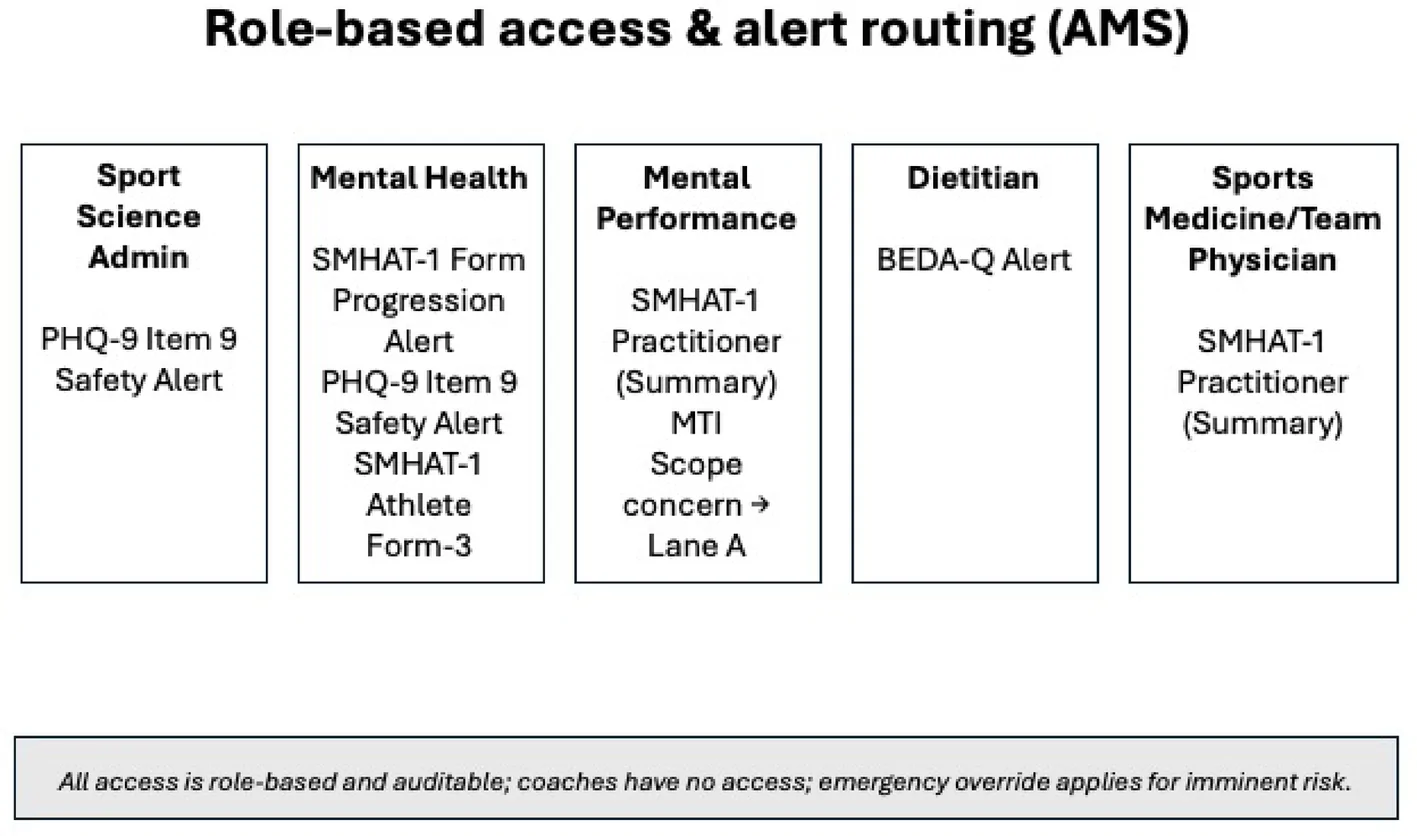
Role-based access and governed alert routing for SMHAT-1 and MTI workflows within an athlete management system (AMS). The AMS applies role-based permissions to govern who can view specific screening outputs and receive alerts. Mental health (MH) clinicians receive SMHAT-1 progression alerts, safety alerts, and access to clinical forms and follow-up documentation. Mental performance (MP) staff access MTI outputs and authorized non-clinical summaries to inform skills-based support within scope. If MP concern exceeds non-clinical scope a warm handoff/secure referral to MH clinician is initiated. Dietitians receive domain-specific alerts relevant to nutrition-related follow-up. Sports medicine and team physicians access practitioner summaries to support medical oversight during pre-participation and ongoing care. A sport science operational administrator may receive routing-status notifications for urgent alerts solely to verify closed-loop escalation and system functioning with clinical interpretation and follow-up retained by licensed providers. All access and actions are auditable within the AMS; coaches have no access; and emergency override procedures are used only when imminent safety concerns require immediate escalation

Governance is addressed in detail in Sect.  3.1 and is introduced here only to clarify the workflow shown in Fig. 1. In brief, the model uses role-based, auditable access and predefined routing so that MH screening outputs are directed to licensed MH or medical providers, MP outputs are directed to qualified MP practitioners within non-clinical scope, and urgent concerns follow institutionally defined escalation procedures. Sport science personnel may support workflow-status monitoring and closed-loop documentation, but they do not interpret MH data, conduct clinical review, contact athletes for clinical follow-up, conduct risk assessment, or substitute for licensed providers. Figure 1 illustrates this role-based access and governed alert-routing logic.

This model is intended to be adaptable across institutional contexts. Specific staffing patterns, software vendors, reporting structures, and external partnerships will vary across institutions, and some components may need to be scaled to local resources and capacity. The core proposition, requiring prospective evaluation, is that MH–MP integration may be safer and more reliable when coordination is governed, role-specific, privacy-secured, auditable, and clinician-led where clinical concerns arise.

### The “two-lane bridge” model

**Fig. 2:**
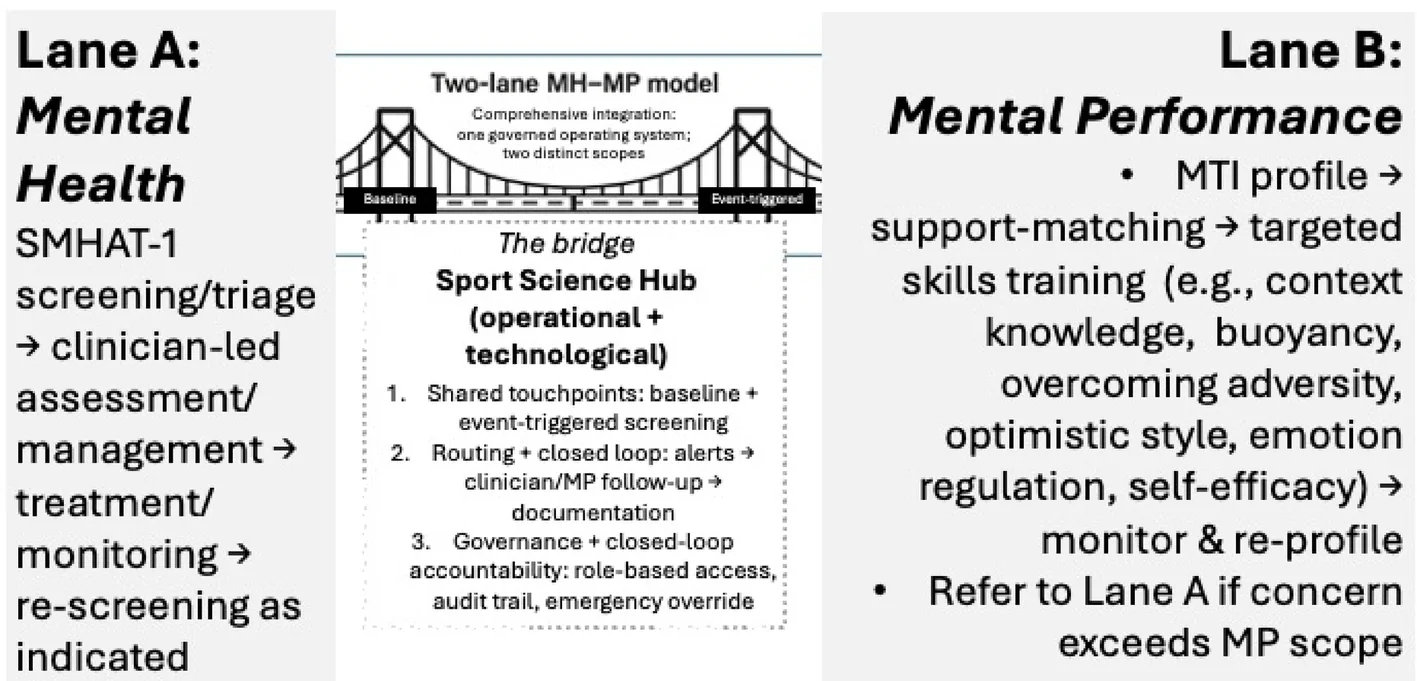
Proposed two-lane MH–MP model within a governed sport science hub structure. Lane A represents the clinician-led mental health (MH) pathway, including structured screening and triage, assessment and management, treatment or referral, monitoring, possible participation restriction when clinically indicated, and individualized reentry when restriction occurs. Lane B represents the non-clinical mental performance (MP) pathway, including applied profiling, support-matching, and targeted skills-based training delivered within scope by qualified MP practitioners; the MTI is one illustrative profiling tool rather than a diagnostic instrument or comprehensive model of MP. When MP concerns exceed non-clinical scope, a warm handoff or secure referral to an MH clinician is initiated. The sport science hub functions as the coordinating bridge by linking shared touchpoints, enabling closed-loop handoffs and documentation, and supporting governance through role separation, auditable routing

We propose a two-lane mental health–mental performance (MH–MP) model (Fig. 2) that intentionally connects workflows while preserving clear purpose and scope boundaries [1, 5]. Lane A is the clinician-led MH pathway, including structured screening, triage, assessment, referral, and follow-up. Lane B is the non-clinical MP pathway, including applied profiling, support-matching, and skills-based support delivered within scope. Its purpose is broader than MT alone and includes performance-relevant psychological competencies such as coping, attentional control, emotional regulation, confidence, motivation, self-regulation, stress management, and preparation routines. The bridge between lanes consists of shared touchpoints, governed handoffs, and escalation pathways that allow concerns arising in one lane to prompt appropriate referral in the other while preserving distinct clinical and performance functions. This two-lane model therefore advances a focused conceptual claim: scalable MH–MP integration in collegiate sport depends on coordinated infrastructure and governed handoffs across distinct but connected services. The sport science hub contributes by connecting the lanes operationally, while licensed clinicians retain responsibility for MH interpretation, risk assessment, and treatment decisions. Within this model, MP practitioners should be trained to recognize signs that exceed non-clinical performance-support scope and to initiate referral to licensed MH providers through predefined pathways.

In the illustrative workflow described here, the MTI serves as one applied profiling tool for support-matching and skills-based intervention within non-clinical scope. This distinction is important because MT remains conceptually and psychometrically debated. Beyond the unidimensional MTI approach, other traditions conceptualize MT through multidimensional or bifactor models, including the 4Cs/6Cs framework and the Sport Mental Toughness Questionnaire, and recent psychometric work has examined whether different instruments measure the same construct or overlapping but distinct psychological resources [47–52]. Accordingly, the MTI is used pragmatically to inform non-clinical support within clear pathways to clinician-led care when concerns exceed MP scope; it does not function as a diagnostic instrument, comprehensive model of MP, or basis for delaying MH referral.

Because Lane B is a non-clinical pathway, the model requires explicit criteria for referral from MP support to clinician-led MH care. Triggers for Lane B → Lane A referral may include disclosure of significant distress, suicidal ideation or self-harm language, marked behavioral or functional change, concerns related to eating, substance use, trauma or abuse, persistent performance disruption not better explained by training, injury, illness, or contextual factors, or any MP session content suggesting possible clinical risk. In these situations, the MP practitioner initiates a warm handoff when possible, or a secure referral to the designated licensed MH provider or clinical lead, with documentation limited to the minimum necessary information. Same-day escalation is used for urgent concerns, and emergency procedures are activated for imminent safety concerns. Responsibility for assessment, diagnosis, treatment planning, and participation-related recommendations remains with licensed clinical providers. Athletes may therefore receive support in both lanes concurrently, but the responsibilities do not merge: clinical interpretation, risk assessment, treatment planning, emergency management, and participation-related recommendations remain in Lane A, while Lane B may continue only as non-clinical skills-based support with athlete consent and, when appropriate, coordination with the clinical provider.

### Implementation layers (how sport science functions as the hub)

**Fig. 3:**
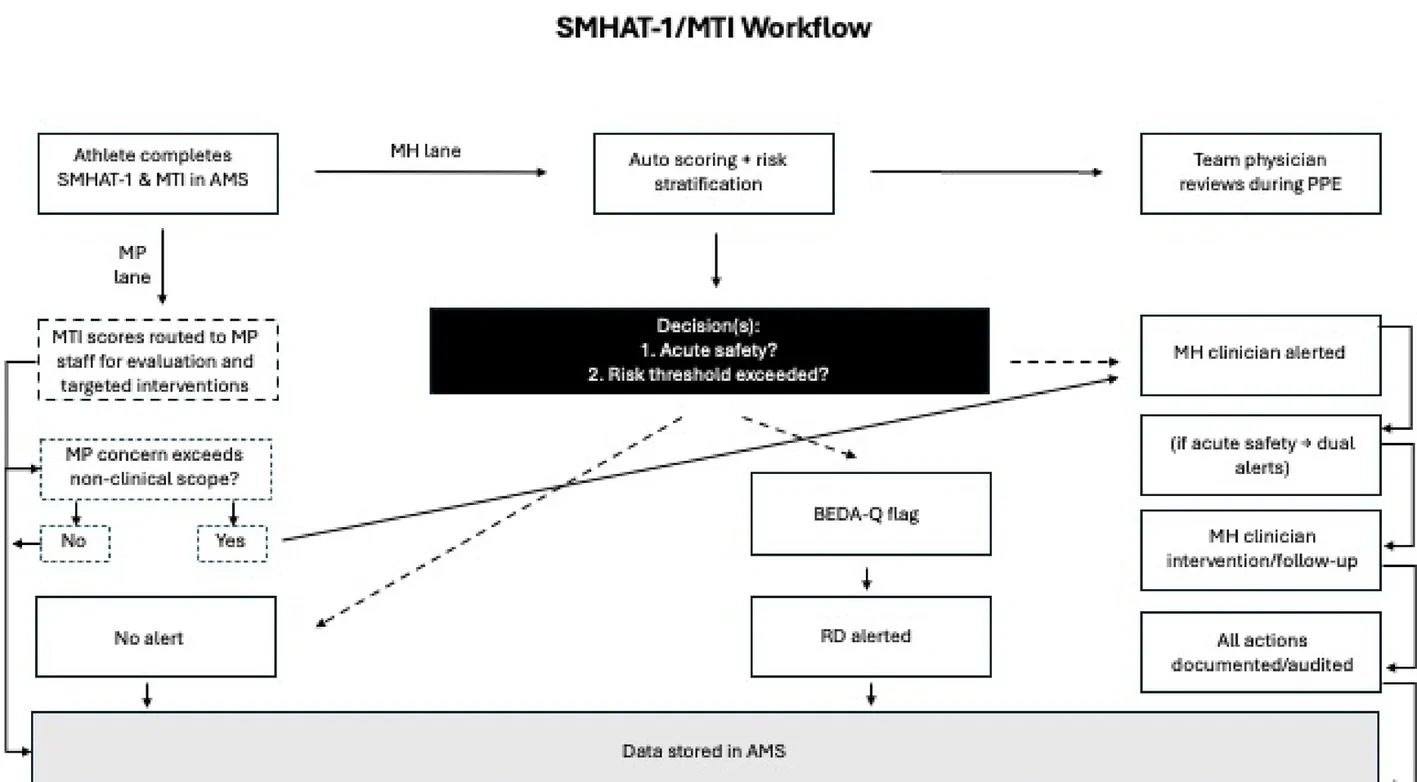
Illustrative integrated MH-MP workflow within an athlete management system (AMS). Student-athletes complete structured MH screening and MP profiling electronically within a privacy-secured platform. The system supports automated scoring, role-based routing, documentation, and auditable follow-up. MH risk thresholds or urgent safety concerns are routed to licensed clinicians or appropriate medical providers for clinician-led follow-up, with escalation procedures activated under institutional policy when needed. MTI outputs may be routed to qualified MP practitioners to inform support-matching and skills-based support within non-clinical scope. Concerns that exceed MP scope prompt a predefined Lane B → Lane A handoff through warm handoff or secure referral, with documentation limited to the minimum necessary information. Domain-specific alerts may also be routed to qualified providers, such as registered dietitians, when relevant. The figure illustrates governed workflow logic that can be adapted to local institutional protocols

This section translates the MH–MP model into five implementation layers that can be adapted across staffing models and software workflows. Sport science may serve as an integration function that helps make governed MH–MP coordination more workable across institutional contexts (Fig. 3).

**(a) Organizational layer: role separation, governed handoffs, and accountability**

NCAA best practices emphasize written action plans that define identification, referral, follow-up, reentry, responsibilities, and qualified provider oversight [1]. Within our model, sport science supports workflow integration, documentation, and system accountability, while clinical authority remains with licensed or otherwise qualified providers. Licensed MH clinicians and sports medicine providers retain responsibility for MH interpretation, risk assessment, referral, and treatment decisions, while qualified MP practitioners provide skills-based support within non-clinical scope and initiate referral to Lane A when concerns exceed that scope [1, 2, 5]. Coaches are positioned as educated supporters who may recognize concern and encourage help-seeking, while clinical screening and alert loops remain limited to appropriate health and medical personnel [1].

The action plan should also define what happens after an athlete enters the MH support pathway, including clinical follow-up, monitoring, possible participation restriction, and reentry when restriction occurs. Entry into this pathway may occur through several routes: a positive screen, athlete self-referral, coach or staff concern, peer concern, or referral from another member of the health and performance team. Entry into MH support does not automatically restrict sport participation, even when the concern is serious or requires same-day clinical follow-up. The licensed MH therapist would typically meet with the athlete and, when safety, risk, or participation concerns are present, consult with the designated medical lead, such as a team psychiatrist or other qualified physician.

If an athlete is temporarily removed from participation, return to participation should follow an individualized timeline. Reentry should be individualized based on ongoing counseling, clinical monitoring, risk assessment, athlete readiness, and, when indicated, psychiatric consultation or treatment. Some cases may include repeated standardized assessment, whereas others may rely primarily on clinical interview, treatment response, and interdisciplinary consultation. The sport science hub or athlete management system may support closed-loop documentation that referral, monitoring, participation restriction, and reentry steps occurred, while readiness, clinical status, and clearance remain clinician-led determinations. MP support may resume or continue within non-clinical scope, with athlete consent and, when appropriate, coordination with the treating MH provider.

**(b) Ethical layer: autonomy**,** consent**,** surveillance**,** and clinical independence**

The governance structure must also account for ethical tensions that are heightened in collegiate sport. Standard ethical assumptions about autonomous consent, confidentiality, and clinical independence are complicated when care is delivered within a sport environment shaped by scholarships, roster status, coach influence, team selection, medical clearance, and performance expectations. In sports medicine, the traditional clinician–patient relationship often becomes a clinician–athlete–team relationship, creating potential conflicts between athlete autonomy, institutional oversight, and team interests [53, 54]. These tensions explain why the operational safeguards described in Sect. 3.1 are not merely administrative features but ethical conditions for implementation.

Consent should therefore be treated as an ongoing communication process. In integrated MH–MP systems, athletes may be able to decline screening, profiling, or data sharing, while also perceiving participation as expected or refusal as consequential. This distinction between formal voluntariness and substantive voluntariness is especially important in sport settings where athletes may fear deselection, reduced playing time, scholarship consequences, or negative perceptions from coaches and staff [55, 56]. To reduce coercive pressure, institutions should separate screening and consent processes from coaching decisions, use plain-language explanations of what is voluntary and what is required, define who can access which data, and provide routes for athletes to ask questions or seek care outside athletics when desired.

Integrated data systems also require attention to perceived surveillance and data-use boundaries. MH information, MP profiles, training data, injury data, and behavioral indicators may have different legal and ethical protections, yet athletes may experience them as part of one institutional monitoring system. Data governance should therefore specify information flow, access permissions, purpose limits, and conditions for sharing across MH and MP lanes. Clinical MH data should remain restricted to appropriate health and medical personnel, and performance data should be interpreted within its intended non-clinical purpose rather than used to infer clinical status or justify punitive decisions. Athlete-facing transparency mechanisms, such as clear data-use statements and, where feasible, access logs or summaries of who has viewed sensitive information, may strengthen trust.

Finally, clinical independence must be protected against the power imbalance between athletics and healthcare. Even when clinicians retain formal authority, coaches, administrators, and performance staff may shape help-seeking through cultural signals, informal expectations, or pressure around availability and participation. The proposed hub model should therefore be understood as one governance response to this structural asymmetry. Its ethical defensibility depends on independent clinical judgment, minimal necessary data sharing, clear separation between care decisions and sport-selection decisions, and ongoing assessment of athlete trust, perceived coercion, privacy concerns, and real-world deviations from policy.

**(c) Technology layer: privacy-secured administration**,** routing**,** and auditability**

A privacy-secured digital platform can support standardized administration of screening and profiling measures, automated scoring, role-based routing, and auditable follow-up [1, 2]. In this model, technology functions as infrastructure: it is intended to help connect identification to action while clinical judgment remains central. Access should be limited to personnel with a legitimate need to know, with coaches excluded from clinical MH data [1]. Where urgent safety concerns are detected, any redundant routing notification to non-clinical operational personnel should support closed-loop workflow verification under institutional policy; clinical interpretation, athlete contact, and emergency management remain the responsibility of licensed clinicians and medical personnel.

**(d) Communication layer: standardized messaging**,** trust**,** and help-seeking**

Implementation is likely to depend, in part, on athlete understanding of why screening occurs, who can view the information, how it will and will not be used, and what happens after a positive screen [1]. Standardized communication should therefore clarify confidentiality limits, non-punitive use protections, routes for urgent help-seeking, and the distinction between MH care and MP support. Clear messaging also matters because self-report responses may be influenced by stigma, trust, and perceived consequences, with implications for underreporting, overreporting, and follow-through [1].

**(e) Partnership layer: campus**,** health system**,** and community coordination**

Integrated MH–MP systems may also depend on external partnerships. In practice, this may include counseling services, psychiatry and psychology networks, academic support, crisis services, and community referral options coordinated through explicit consent processes, institutional policy, and local legal and administrative frameworks [1, 5]. These partnerships should be understood as governed extensions of the workflow rather than informal add-ons, especially when referral completion, crisis response, or cross-system data sharing is required. Because institutions differ in staffing and resources, these partnerships are likely to require local adaptation, and less-resourced settings may need to scale implementation accordingly.

Together, these five layers clarify the central claim of this article: sport science may be useful as one coordinating infrastructure for governed handoffs, follow-through, and accountability, with clinical expertise, local adaptation, and athlete trust remaining central.

## Guardrails, practical steps, and a forward-looking research agenda

### Core safeguards and governance guardrails

This framework is organized around several explicit guardrails. First, screening is not diagnosis. Structured screening tools can support earlier recognition and triage, but positive screens require clinician-led assessment and follow-up, and negative screens do not eliminate the possibility of clinically meaningful concern [1–4]. We use SMHAT-1 as an example of a structured pathway because integrated systems may benefit from a governed mechanism for connecting screening to action, with screening results interpreted within broader clinical workflows [2–4].

Second, MH care should remain clinician-led and within scope. Licensed or otherwise qualified MH and medical professionals are responsible for interpretation of screening results, risk assessment, referral, treatment decisions, emergency management, participation restriction, and reentry decisions [1, 2]. Positive screens, referrals, disclosures, or MP concerns should lead to individualized participation-related decisions that are clinically justified and documented through the action plan.

Third, MP profiling is non-diagnostic. In this Perspective, the MTI is treated as one applied index that may help organize support priorities within the MP lane, with its use limited to non-clinical support planning rather than diagnosis, comprehensive MP assessment, or clinical subscale interpretation. This caution is especially important because MT is conceptually and psychometrically debated across multiple frameworks and instruments [27, 47, 48, 50–52]. Any item-level labels used in applied settings function as pragmatic aids for support targeting.

Fourth, performance concerns must be interpreted in context. Unexplained performance disruption may sometimes warrant consideration within a governed screening or referral workflow, particularly when persistent and not better accounted for by injury, illness, training load, technical factors, or situational stressors. Performance fluctuation alone should be interpreted within a broader clinical, performance, and contextual assessment rather than as evidence of psychopathology [1, 2].

Fifth, trust protections and privacy safeguards are central considerations for implementation. Screening information should not be used for selection, playing time, scholarship status, or team standing, and access must be role-based, auditable, and limited to personnel with a legitimate need to know [1]. Coaches may play an important role in education, recognition, and support, while clinical screening and alert loops should remain limited to appropriate health and medical personnel.

Sixth, urgent escalation should remain clinician-led. Non-clinical operational personnel may support workflow monitoring only in a narrowly defined, auditable, closed-loop capacity under institutional policy. Clinical interpretation, licensed follow-up, and clinical decision-making remain outside the role of non-clinical operational personnel. Emergency response, athlete contact, safety planning, and referral remain the responsibility of licensed clinicians and medical personnel.

Finally, governance mechanisms require ongoing evaluation in practice. Role-based access, audit trails, consent procedures, and non-punitive use protections are necessary structural safeguards, but their effectiveness depends on how athletes perceive and experience the system in practice. Governance should therefore include recurring athlete-facing education, plain-language consent and confidentiality explanations, mechanisms for athlete feedback, monitoring of trust and acceptability, review of protocol deviations, and continuous improvement processes that examine whether the system is experienced as safe, voluntary, non-punitive, and clinically appropriate.

### What departments can do now: a minimal viable approach to MH–MP integration

A minimal viable model may begin with a governed foundation before full institutional complexity is developed.

1. Start by building the bridge before building advanced analytics. Establish written workflows for screening, triage, referral, documentation, and emergency escalation before adding dashboards, predictive models, or cross-domain decision support [1, 5].
2. Use a structured screening and referral pathway to limit reliance on ad hoc communication. Institutions may choose different instruments, but the core principle is that screening should be connected to qualified follow-up, defined responsibilities, and documented handoffs [1, 2].
3. Create clear lane separation with explicit handoff rules. This includes defining what belongs in the clinician-led MH lane, what belongs in the non-clinical MP lane, and what events may warrant governed referral across lanes.
4. Clarify consent, privacy, and non-punitive use protections in plain language. Athletes need clear information about why screening occurs, who can view the information, what confidentiality limits apply, and how the information will and will not be used [1].
5. Monitor a small set of system-performance indicators from the beginning. Useful early metrics include screening uptake, positive-screen volume, time to clinician contact, referral completion, re-screening cadence, and athlete trust or acceptability indicators. At this stage, these measures may provide a practical foundation for later analytic or predictive work.
6. Scale the model to local resources and partnerships. Multi-layered governance, rapid response pathways, and protected access structures will vary across resource settings. For that reason, this model is presented as a governed illustrative coordination model that can be adapted to local staffing, campus resources, and community referral capacity.

### **Research agenda for evaluating integrated MH–MP infrastructure**

A governed MH–MP infrastructure raises several testable research questions aligned with the article’s framing and preliminary implementation observations.

1. Implementation science. Future studies could examine acceptability, athlete trust, stigma, privacy perceptions, clinician workload, workflow fidelity, referral completion, and unintended consequences, including the risk of misinterpretation by non-clinical stakeholders.
2. Screening performance and decision quality. Prospective work could evaluate false-negative and false-positive consequences, burden on referral systems, and whether event-triggered screening improves case detection without eroding trust [3, 4].
3. Prospective MH–MP relationship testing. If MP indicators are to inform future support planning, they should be studied in preregistered designs with appropriate adjustment for confounders and with explicit testing of both linear and non-linear possibilities.
4. Care coordination across systems. Integrated models could be evaluated for how effectively they coordinate with campus counseling, psychiatry, sports medicine, academic support, and community providers under consent and local legal or administrative frameworks.
5. Stakeholder-informed refinement. Athlete perspectives, practitioner feedback, and attention to cultural context, education, and equity should be considered into future model development and evaluation, particularly as institutions differ in resources, staffing, and climate. Future work should also examine how athletes interpret MTI-informed profiling across gender, cultural, and sport contexts, including whether it is experienced as useful, non-punitive, and distinct from cultural toughness norms that discourage vulnerability or help-seeking [40, 45, 46].

Taken together, these priorities frame the central argument of this article as a set of implementation hypotheses: governed infrastructure may help connect recognition to action in ways that are ethical, trusted by athletes and stakeholders, and sustainable, but these assumptions require prospective evaluation.

## Conclusion

Recent NCAA and IOC guidance underscores a practical reality for collegiate sport: mental health support is likely to be more effective when structured, non-punitive, and connected to timely follow-up through coordinated processes [1, 2]. In that context, infrastructure may be important for translating awareness into action. In this Perspective, we propose that a sport science hub can serve as one possible coordinating architecture for linking clinician-led MH screening, triage, referral, and follow-up with non-clinical MP profiling and skills-based support, while preserving role separation, privacy protections, and athlete trust [1, 2, 5].

The model is offered as a candidate framework for coordinated MH–MP infrastructure across collegiate sport settings rather than a validated blueprint. The framework has been operationalized in one collegiate sport setting, with preliminary local observations reported separately as conference abstracts [57, 58], but this experience is offered only as implementation context rather than evidence of effectiveness, generalizability, superiority, predictive validity, or decision thresholds. Its intended contribution is to clarify how institutions might move from fragmented service availability to more coordinated, auditable, and ethically governed MH–MP systems.

Future work should evaluate whether governed MH–MP coordination is associated with improvements in follow-through, acceptability, screening performance, referral completion, care coordination, and athlete trust across settings. The central proposition of this Perspective is not that governed integration has already been validated, but that better-governed systems may help connect recognition to action in ways that are ethical, trusted, and sustainable.

## Data Availability

No datasets were generated or analysed during the current study.
